# Editorial: Anaphylaxis challenges: idiopathic and rare causes

**DOI:** 10.3389/falgy.2026.1804294

**Published:** 2026-03-27

**Authors:** Maryam Ali Al-Nesf, Hassan Mobayed, Michael Makris, Iman Nasr, Ivan Cherrez-Ojeda, Luisa Ricciardi

**Affiliations:** 1 Allergy and Immunology Division, Department of Medicine, Hamad Medical Corporation, Doha, Qatar; 2 Allergy Unit, 2nd Department Dermatology and Vevereology, University General Hospital ‘Attikon”, Medical School, National and Kapodistrian University of Athens, Athens, Greece; 3 Immunology and Allergy Unit, Royal Hospital, Muscat, Oman; 4 Espíritu Santo University Allergy and Pulmonology Department, Samborondón, Ecuador; 5 Institute of Allergology Charite Universitatmedizin Berlin, Corporate member of Freie Universitat Berlin and Humboldt-Universitat zu Berlin, Berlin, Germany; 6 Operative Unit of Allergy and Clinical Immunology, Department of Clinical and Experimental Medicine, University of Messina, Messina, Italy

**Keywords:** allergic reactions, anaphylaxis, diagnostic challenges, idiopathic anaphylaxis, rare causes, systemic reactions

## Introduction

The impact of anaphylaxis extends well beyond the acute allergic episode, imposing a substantial burden on patients, clinicians, and healthcare systems. From patients' perspective, anaphylaxis is not defined solely as an acute reaction, but also as a persistent fear of recurrence and the uncertainty surrounding future exposure. Consequently, anaphylaxis greatly impacts quality of life, shaping dietary choices, social interactions, travel, employment, or schooling, and psychological wellbeing (1). The burden is particularly significant when the causative factor cannot be clearly identified, cannot be reliably avoided, or when multiple interacting cofactors such as exercise, infections, medications, or stress contribute to disease expression leaving patients and their family in a continuous state of vigilance and lifelong dependence on adrenaline auto-injectors (2).

These patient-level challenges are mirrored in daily clinical practice. In many cases of anaphylaxis, the inability to confirm a final diagnosis following extensive investigation makes anaphylaxis one of the most challenging conditions encountered in allergy practice. Physicians are frequently confronted with diagnostic uncertainty arising from heterogeneous clinical presentations, limited availability and/or access to sensitive biomarkers, and in some clinical scenarios overlap with other acute medical conditions. These difficulties are further amplified in idiopathic anaphylaxis, cofactor-dependent reactions, and reactions triggered by rare or unconventional allergens, where classical IgE-mediated mechanisms do not fully explain disease expression.

Real-world insights into uncommon presentations, diagnostic pitfalls, and emerging management strategies provided the foundation for the Research Topic “*Anaphylaxis Challenges: Idiopathic and Rare Causes*.” By focusing on Idiopathic reactions, unusual triggers, and atypical phenotypes, this Research Topic aims to advance understanding, support clinical decision-making, inform future research, and subsequently improve outcomes for patients living with this high-risk condition. This topic also aimed to investigate geographical diversity, cultural dietary habits, environmental exposures, and healthcare inequalities that may further contribute to variability in anaphylaxis recognition and management.

The resulting collection comprises twelve peer-reviewed contributions, including case reports, mini-reviews, and original research articles, offering complementary perspectives on idiopathic anaphylaxis, rare allergens, pediatric disease evolution, biologic therapies, desensitization strategies, and life-threatening refractory reactions.

## Topics addressed in the collection

### The role of cofactors in idiopathic anaphylaxis

Anaphylaxis continues to represent the most severe spectrum of allergic reactions and may be life-threatening if not recognized or treated early (3, 4). The burden of anaphylaxis is increasing worldwide, affecting patients across all age groups. Idiopathic anaphylaxis remains a diagnosis of exclusion and continues to pose significant challenges for clinicians, patients and health systems. Idiopathic reactions may be driven by underrecognized cofactors rather than the absence of a trigger. These include physical exertion, infections, medications, hormonal influences, emotional stress, and environmental conditions.

Within this Research Topic, contemporary perspectives emphasize that idiopathic anaphylaxis should be viewed as a dynamic condition, requiring repeated clinical reassessment rather than a static diagnosis. Recognition of cofactors is essential for improving prevention strategies, patient education, and long-term outcomes.

Elkhalifa et al. discussed idiopathic anaphylaxis describing it as unpredictable, reported more frequently in women, and with a wide age range of onset. Female sex in general has been well established as a risk for anaphylaxis particularly from puberty through middle age. This is likely due to estrogen increasing nitric oxide production, augmenting vascular leakage (5), and Progesterone hypersensitivity reactions around the menstruation (6).

Exercise-induced anaphylaxis (EIA) and food-dependent exercise-induced anaphylaxis (FDEIA) represent illustrative examples of cofactor-dependent anaphylaxis. The potentiality of exercise as a possible cause of anaphylaxis, together with other trigger factors such as heat and humidity, must always be kept in mind when taking the patients' history, after an anaphylactic reaction. Mohamed et al. describe a case report of a 20-year-old woman who experienced wheat-dependent-exercise-induced-anaphylaxis (WDEIA) which responded to omalizumab, an anti-IgE biological treatment, administered with a primary indication of prophylactic treatment of WDEIA. Omalizumab treatment was also investigated by Mobayed et al. to avoid anaphylaxis induced by strenuous exercise and/or the exposure to heat and humidity. In general, biologic treatment particularly Omalizumab may represent a promising option in selected patients including those receiving venom immunotherapy (7) and those with refractory anaphylaxis (8).

### Rare allergen exposures

Several reports in this Research Topic illustrate anaphylaxis triggered by unconventional sources, including traditional herbal medicines and insect ingestion. Pan et al. described a case of pollen food allergy syndrome induced by Sua Zao Ren, which is a traditional Chinese medicine, and, as described from the authors, can cause allergic reactions in patients sensitized through pollinosis. A rare case of anaphylaxis from ingestion of Polistes olivaceus larvae was described by Maillot et al. Food additives (reported elsewhere/ not part of this collection), particularly carmine, that are extracted from insects was reported in patients with unexplained anaphylaxis (9) provided more support of human allergenicity to ingested insects. The recognition that insect ingestion can cause allergic sensitization has become a topic of growing interest, particularly with the increasing commercialization of insect flour and other insect-containing food products globally.

### Drug-associated reactions and developing desensitization protocols

Venom immunotherapy can cause anaphylaxis during treatment course, yet severe systemic with particular cardiovascular manifestations are not common. Brunetto et al. described a case of anaphylaxis after administration of a maintenance dose of Hymenoptera venom immunotherapy, with preeminent cardiac involvement evidenced by ECG alterations disappearing after the patient's recovery from the anaphylactic reaction. A possible underlying mast-cell disorder was suggested to trigger such unexpected anaphylactic reaction.

The rapid expansion of novel pharmaceuticals as well as biologic therapies has introduced new hypersensitivity challenges. Several contributions demonstrate how structured desensitization protocols can enable continued access to essential medications in patients who have experienced severe allergic reactions. Said et al. developed and tested a desensitization protocol to insulin, while Isaac et al. successfully desensitized patients to Volanesorsen; the only existing therapeutical option for treating Familial chylomicronemia syndrome (FCS). These articles illustrated that immunological principles of tolerance induction remain fundamental in allergy practice and can continue to grow across diverse therapeutic classes.

Moreover, Aqel et al. described real-world experience in evaluating patients with allergic reactions to COVID-19 mRNA vaccines. Diagnostic evaluation of polyethylene glycol and polysorbate 80 hypersensitivity remains complex, yet structured work-up pathways through skin prick and intradermal testing and multidisciplinary collaboration can facilitate safe vaccination without compromising public health efforts.

### Pediatric anaphylaxis and disease evolution

Children represent a uniquely vulnerable population in whom anaphylaxis may reflect evolving immune mechanisms. Original research by Tran et al. demonstrated the progression from non-IgE-mediated food allergy in infancy to persistent IgE-mediated disease in 5 cases of infants who developed severe, persistent IgE-mediated cow's milk allergy as a sequela of food protein-induced allergic proctocolitis, highlighting the dynamic nature of allergic sensitization.

Melethil and Yousef added a complementary review addressing rare pediatric allergens causing anaphylactic shock related to food ingestion in children highlighting possible hidden causative agents and the importance of long-term follow-up, anticipatory guidance, and age-specific diagnostic strategies to reduce morbidity and improve quality of life.

### Severe and refractory anaphylaxis

At the extreme end of the clinical spectrum lies refractory anaphylaxis, which remains associated with significant mortality. Grafeneder et al. presented a very unusual case report of a multi-phasic life-threatening anaphylaxis refractory to epinephrine treatment who responded to extracorporeal membrane oxygenation (ECMO) illustrating the potential role of advanced life-support modalities when conventional treatment fails, prompt escalation of care, and integration between allergy specialists, emergency physicians, and intensive care teams.

## Conclusion

Conclusively, this Research Topic brings together diverse yet interconnected perspectives on idiopathic and rare causes of anaphylaxis. By integrating case-based observations with broader conceptual insights, the collection advances understanding of anaphylaxis as a multifactorial, dynamic, and highly individualized condition.
